# Importance of abdominal circumference and body mass index values in predicting male hypogonadism – A practical approach

**DOI:** 10.1590/2359-3997000000203

**Published:** 2016-08-31

**Authors:** Paulo Eduardo Dietrich Jaworski, Anderson Ramos, Arthur Radaelli Nicoleit, Luis Fernando de Almeida Bacarin, Pedro Olivo

**Affiliations:** 1 Departamento de Urologia Hospital Universitário Evangélico de Curitiba Curitiba PR Brasil Departamento de Urologia, Hospital Universitário Evangélico de Curitiba, Curitiba, PR, Brasil; 2 Faculdade de Medicina Faculdade Evangélica do Paraná Curitiba PR Brasil Faculdade de Medicina, Faculdade Evangélica do Paraná (Fepar), Curitiba, PR, Brasil

**Keywords:** Abdominal, circumference, obesity, testosterone

## Abstract

**Objective:**

The objective of this study was to correlate the values of abdominal circumference (AC) and body mass index (BMI) with the levels of total testosterone (TT), free testosterone (FT) and sexual hormone binding globulin (SHBG). We aimed to analyze the association between the anthropometric values and variations in the hormonal levels.

**Subjects and methods:**

A transversal prospective study was conducted. A total of 159 patients were included in the study.

**Results:**

BMI was inversely correlated with TT and SHBG (p = 0.02 and p = 0.006, respectively). AC was also inversely correlated withTT and SHBG (p = 0.006 and p < 0.0001, respectively). However, BMI did not correlate signicantly with these hormonal levels after adjusting for age.

**Conclusion:**

This finding led to the conclusion that AC had a stronger inverse correlation than BMI with TT and SHBG. Our data suggested that AC alone can be used as an anthropometric parameter to help simplify the identification of men with low serum TT levels.

## INTRODUCTION

Population aging has widespread consequences on all sections of organized civil society. From a medical perspective, clinicians are called upon to address the prevention, treatment and even retardation of the consequences of aging. Aging women experience a decrease in estrogen which invariably evolves into menopause, a very well-known condition that is often discussed by doctors and patients. Aging men usually develop a decrease in androgen levels ( 1 ). These biochemical findings and the associated specific symptoms are known as Androgen Deficiency of the Aging Male (ADAM). The array of associated symptoms include decreased libido, erectile dysfunction, decreased ejaculate volume, hair loss, weakness, and anemia ( 2 ). Low testosterone levels can also lead to decreased bone mineral density and lean mass, increased fat mass, and increased cardiovascular risk including atherosclerosis and the metabolic syndrome ( 3 - 6 ).

Despite being the subject of many studies, male hypogonadism and ADAM are not easily identified during office consultations, due to cultural and even social characteristics of Brazilian males, particularly among the elderly. A method to effectively identify these patients is necessary in order to expand the multidisciplinary treatment of the aging male population, with the aim to improve their global quality of life.

The objective of this study was to analyze two anthropometric values, abdominal circumference (AC) and body mass index (BMI), and compare them to serum levels of total testosterone (TT), free testosterone (FT) and sexual hormone binding globulin (SHBG). We aimed to identify the best anthropometric-hormonal correlation that could help identify patients with a potential diagnosis of ADAM.

## SUBJECTS AND METHODS

A transversal prospective study was conducted. We included male outpatients in our service who were greater than 40 years old in a random manner, without distinction by race or ethnicity. Patients were excluded if any other possible cause of hypogonadism was identified during the consultation ( *e.g* ., use of medication, testis affections, hyperprolactinemia). All of the subjects signed an informed consent form. A total of 159 men agreed to participate in the study. The institutional Ethics Committee approved the study.

The data collection took place over 4 months, between April and August of 2013. The anthropometric data (height, weight and abdominal circumference) and age were measured in a preliminary interview. Each subject had a fasting blood sample drawn between 8:00 h and 10:00 h. Serum levels of total testosterone, free testosterone and SHBG were analyzed.

The total testosterone and SHBG levels were measured using chemiluminescence immunoassay (CLIA) kits, and the free testosterone was calculated according to Vermeulen’s Formula (http://www.issam.ch/freetesto.htm). Height was measured in meters (m) and weight in kilograms (kg), and both measurements were obtained with the subject barefoot and wearing only underpants. The AC was measured in centimeters (cm), using the landmark of the average point between the 10^th^ rib and the iliac crest at the axillary line. The subjects were divided into two groups, patients with an AC higher than 102 cm and patients with an AC lower than 102 cm, based on the AC criteria for patients at high risk for metabolic disease ( 7 ). The BMI was calculated and patients were also divided in two groups, patients with a BMI higher than 30 and patients with a BMI lower than 30, based on the World Health Organization criteria for obesity classification ( 8 ). The data were organized in a frequency and contingency table. The Kolmogorov-Smirnov test was used to determine the sample distribution. The central tendency was displayed as the average and standard deviation for parametric data, whereas the median and interquartile intervals were used for non-parametric data.

The AC and BMI were individually correlated with TT, FT and SHBG levels using the Spearman test. Multiple linear regression was used to analyze independent variables and to correlate age, BMI and AC. The ANCOVA was used to analyze correlations between hormonal values and the 4 groups of subjects, according to their BMI (higher than 30, lower than 30) and AC (higher than 102 cm, lower than 102 cm).

All the statistical tests were deemed significant at a p-value lower than 0.05. The analytical software utilized was MedCalc v.10.0.

## RESULTS

The demographic data are displayed in Table 1 . The median age was 66.7 years. The average BMI was 28.1 kg/m^2^ and the average AC was 98.7 cm.

**Table 1:** Demographic and hormonal data

**N**	**159**
Age	66.7 (58.4 – 71.9)*
BMI	28.1 (± 4.3)^#^
AC	98.7 (±11.46)^#^
Total testosterone	369 (291 – 423.6)*
Free testosterone	6.66 (5.17 – 8.28)*
SHBG	39.1 (30.8 – 50.8)*

* Median (inter-quartile interval); ^#^ Average (standard deviation).

BMI was inversely correlated with TT and SHBG, both significantly, but the correlation between BMI and FT was not significant. AC was also inversely correlated with TT and SHBG and these results were with statistically significant. The data are shown in Table 2 .

**Table 2:** Anthropometric measurements and hormonal correlations*

	FT	TT	SHBG
BMI	0.809	0.03	0.006
AC	0.895	0.006	< 0.001

* Spearman’s p-value.

An age-adjusted linear regression analysis demonstrated correlation between the anthropometric parameters and hormonal levels, and the results are shown in Table 3 . We tried to refine our analysis, knowing that the age range could bias the results. Again, we confirmed that BMI and AC both correlated with TT and SHBG.

**Table 3:** Adjusted partial correlation* between anthropometric measurements and FT, TT and SHBG

	Abdominal circumference (AC)
**Free testosterone**	
Age adjustment	P = 0.419
AC adjustment	-
Age and BMI adjustment	P = 0.59
**Total testosterone**	
Age adjustment	P = 0.002
Age and AC adjustment	-
Age and BMI adjustment	P = 0.019
**SHBG**	
Age adjustment	P < 0.001
AC adjustment	-
Age and BMI adjustment	P < 0.001

* Multiple linear regression p-value (CI = 95%).

The group analysis, displayed in Table 4 , revealed that men with an AC lower than 102 cm had significantly lower TT and SHBG levels than men with an AC higher than 102 cm. When the BMI groups were compared, we found that men with a BMI lower than 30 had significantly higher SHBG levels than men with a BMI higher than 30. No significant differences were observed between the groups when TT and FT were analyzed.

**Table 4:** Mean free testosterone, total testosterone and SHBG in different anthropometric subgroups

	AC < 102 cm			AC > 102 cm			p-value*
	
	N	Mean	SD	N	Mean	SD	
Free testosterone	101	7.35	0.31	58	6.96	0.41	0.324
SHBG	101	44.61	1.47	58	37.29	1.94	0.001
Total testosterone	101	394.69	13.7	58	342.74	18.2	0.007

	**BMI < 30**			**BMI > 30**			p-value*

	**N**	**Mean**	**SD**	**N**	**Mean**	**SD**	

Free testosterone	47	7.51	0.22	112	6.88	32.17	0.753
SHBG	47	48.08	1.35	112	37	0.67	0.004
Total testosterone	47	411.4	8.87	112	360	2.36	0.09

* ANCOVA’s p-value.

## DISCUSSION

Men’s testosterone synthesis decreases with age, whereas central obesity tends to increase ( 1 , 9 ). Many authors have demonstrated the existence of an inverse correlation between total testosterone and central obesity when they are analyzed in the same subject ( 10 - 12 ). The proposed pathophysiology is that visceral fat is associated with lower plasmatic SHBG concentration ( 13 , 14 ), which results in more testosterone in a free form, resulting in negative feedback to the pituitary gland, which inhibits the production luteinizing hormone and lowers its level. Therefore, the low SHBG levels decrease testosterone production ( 14 ). Moreover, abundant cytokine production within the fat tissue also inhibits testosterone production through intracellular mechanisms. Alpha tumor necrosis factor (α-TNF), for instance, inhibits steroid production inside Leydig cells, at the intracellular transcription level, whereas interleukine-1 (IL-1) inhibits the cholesterol side-chain cleavage by the p450 cytochrome inside the Leydig cells ( 15 ).

Central obesity and increased abdominal circumference are conditions that correlate to each other. Increased abdominal circumference has been described as the best indicator of central obesity ( 14 ), total body fat ( 16 ) and visceral fat ( 17 , 18 ), particularly in men who are more than 40 years of age. When the production of androgenic hormones in these groups of patients was examined, Svartberg and cols. concluded in the Tromsø study that abdominal circumference values were inversely associated with serum levels of TT, FT and SHBG ( 11 ). In addition, they reported a higher strength of association between abdominal circumference and TT, FT and SHBG when compared to the association between BMI and TT, FT and SHBG. The investigators proposed that, within that patient population, the simple measurement of AC could be useful to anticipate levels of serum testosterone ( 19 ). Similarly, Seidell and cols. have shown that men with higher visceral fat values have lower total and free testosterone serum levels ( 10 ).

Multiple transversal and longitudinal studies ( 11 , 20 , 21 ) have shown that aging men experience progressive declines in their TT and FT levels. This down variation is physiological ( 11 , 20 ), and it is caused by a reduction in testicular function associated with a reduction in the activity of the hypothalamic-pituitary axis ( 22 , 23 ). By adjusting our data for age, we reduced the chance of an eventual distortion in the comparison of the variables and men in different ages. We found that, even after this adjustment, our results remained consistent and statistically significant.

There is controversy in the literature about the cause and effect theory of low androgen levels and obesity. Some authors have suggested that obesity has a causal role in the decline of androgen levels. They state that weight loss can partially reverse low TT and SHBG levels ( 24 - 26 ). However, Khaw and Barrett-Connor showed that TT and SHBG were inversely correlated with visceral fat and age, suggesting that testosterone can mobilize abdominal fat deposits ( 13 ). During the 15 observation years of The Massachusetts Male Aging Study ( 27 ), there was evidence that low testosterone and SHBG levels could predict the incidence of the metabolic syndrome. Ding and cols. concluded that low SHBG levels can predict the risk of developing type 2 diabetes mellitus ( 28 ).

Within our pool of subjects, AC was inversely correlated with TT and SHBG levels. Moreover, AC correlated in a stronger and more consistent manner with TT and SHBG than BMI. Because we recruited a considerable number of men with a heterogeneous age profile and preformed their serum sample analysis in the same laboratory and during the same time interval of 2 hours in the morning, we eliminated diurnal variability in the testosterone levels and provided reliability to our data and results. The limitations of this study include the different sizes of the AC and BMI groups, the limited number of participants and the fact that all of the subjects were recruited from a tertiary urology outpatient clinic, so our findings may not be generalizable to other patient populations.

In conclusion, in this study we found that abdominal circumference had a stronger inverse correlation with TT and SHBG than body mass index. A measured abdominal circumference of greater than 102 cm can be used as anthropometric data to predict low TT and SHBG levels in patients evaluated in a medical office. This simple information can contribute to the correct identification of androgen deficiency symptoms in aging men.
